# Evaluating the prospective crown-root ratio after extrusion and crown lengthening procedures in vitro

**DOI:** 10.1038/s41598-023-46354-y

**Published:** 2023-11-02

**Authors:** Maria Bruhnke, Isabelle Voß, Guido Sterzenbach, Florian Beuer, Michael Naumann

**Affiliations:** Present Address: Department of Prosthodontics, Geriatric Dentistry and Craniomandibular Disorders, Charité – Universitätsmedizin Berlin, corporate member of Freie Universität Berlin, Humboldt – Universität zu Berlin and Berlin Institute of Health, Aßmannshauser Straße 4-6, 14197 Berlin, Germany

**Keywords:** Oral anatomy, Dental anthropology, Dental trauma, Dental treatments, Endodontics, Orthodontics, Prosthetic dentistry, Restorative dentistry

## Abstract

For restoration of extensively damaged teeth preprosthetic treatment measures are necessary. Crown lengthening and extrusion affect the prospective crown-root ratio (CRR). The subject of this in vitro study was to compute CRRs for both treatment approaches. 120 human maxillary central extracted incisors were measured. Measurements were calculated for five treatment groups: C (control), E-2 mm (extrusion of 2 mm), E-4 mm (extrusion of 4 mm), CL-2 mm (crown lengthening of 2 mm), and CL-4 mm (crown lengthening of 4 mm). Tooth (TL), root (RL), and crown lengths (CL) were measured from mesial (m) and facial (f) cemento-enamel junction (CEJ), and respective anatomic (CRR) and effective crown-root ratios (eCRR) were calculated. Following CRR values were computed for C: CRR-m = 0.4 ± 0.1, CRR-f = 0.7 ± 0.1. All crown-root ratios were lower (more favourable) for extrusion compared to crown lengthening (*p* < 0.001). ECRRs were higher than anatomic CRRs. CRR at mesial CEJ was significantly lower than CRR with facial CEJ as reference (*p* < 0.001). Mesial measurement-based calculations of CRR typically based on radiographic images should be interpreted with caution as they underestimate the eCRR. CRR can be expected as lower, i.e. more favourable, when teeth are extruded than crown lengthened.

## Introduction

The restoration of severely destroyed teeth when the clinical crown is lost is challenging. Tooth preservation with endodontic treatment, post-and-core, and crown restoration versus tooth extraction and subsequent implant placement have to be carefully considered and weighed up as equitable treatment alternatives^1^. As concrete clinical guidelines are not available dental practitioners tend to decide differently^2^. Due to alveolar bone loss after extraction^3^ implant placement is in particular in the esthetic zone of the maxillary anterior region regarded as a highly complex procedure^4^. Moreover, implant borne restorations are cost-intensive^5^ and post-operative complications such as perimucositis and periimplantitis are considered downsides for implant placement^6^.

**Figure 1:**
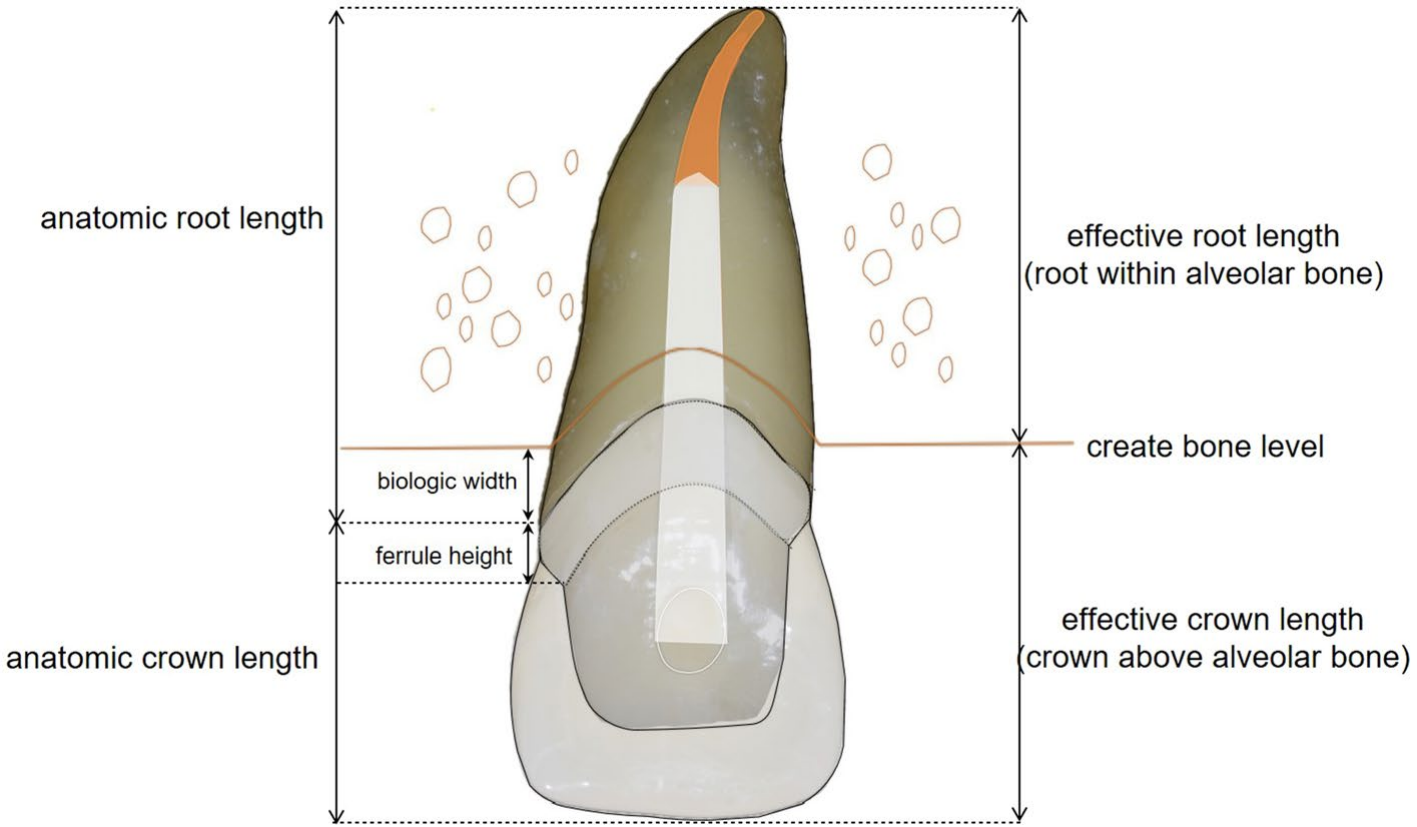
Definition of variables “anatomic crown length”, “anatomic root length “, “effective crown length”, and “effective root length”.

In case of tooth restoration of severely compromised teeth many factors play a role in the treatment planning process: level of defect extension, apical condition of the tooth, tooth mobility score, attachment level, pocket probing depths, quality of endodontic filling, and the prospective crown-root ratio^7–9^. For long-term success of the restoration re-establishment of biologic width^10^ and a circumferential ferrule-design preparation^11^ are deemed necessary. Therefore, preprosthetic therapy options such as crown lengthening^12^ or extrusion^13–17^ have been suggested in literature for preservation and restoration of severely compromised teeth. Surgical crown lengthening is an operative procedure where surrounding bone level is reduced and “the extent of supragingival tooth structure is increased for restorative purposes”^18^. It is connected with an inevitable lengthening of the clinical crown in the same amount as the root will be less supported by surrounding alveolar bone^12^. Particularly in the esthetic zone this procedure may be disadvantageous, since the marginal contour will be disturbed. In contrast, extrusion is the occlusal movement of teeth “beyond the natural occlusal plane that occurs without accompanied movement of their supporting tissues”^18^. Extrusion allows placement of restorative margins above the gingival level re-establishing biologic width^13^. Extrusion may be achieved by orthodontic movement of the tooth^16^ as well as surgical extraction and replantation^19^. Crown lengthening and extrusion procedures affect the prospective crown-root ratio^18^. While for crown lengthening both crown and root lengths are altered, for extrusion only the root length within alveolar bone is reduced. The crown length remains constant. The crown-root ratio is defined as “the physical relationship between the portion of the tooth within the alveolar bone compared with portion not within the alveolar bone, as determined radiographically”^18^. Thereby it is important to differentiate among measurements of the anatomic and the effective crown length (Fig. 1): the anatomic crown is measured from the cemento-enamel junction (CEJ) to the incisal edge^18^ and does not provide information about the alveolar bone support. Therefore, the effective crown length is the portion of the tooth above the alveolar bone, while the effective root length is the portion within the alveolar bone^20^. In literature the crown-root ratio is classified by relatively vague terms such as “favourable”, “unfavourable”, “poor” and “unsatisfactory”^21^. Shillingburg et al. proposed a ratio of 1:1.5 as ideal and a ratio of 1:1 as minimum for abutment teeth^22^. However, both in vitro and clinical evidence to support this rule on definitive proportions is scarce^23–25^. Additionally, there are only few studies available that investigate the biomechanical impact of the crown-root ratio after extrusion in comparison to crown lengthening procedures^20,26,27^.

Therefore, this investigation aimed to calculate crown-root ratios with the example of maxillary central incisors after extrusion and crown lengthening procedures according to clinical demands. Null hypotheses tested were, that there is no difference in anatomic or effective crown-root ratio after simulated extrusion and crown lengthening, and irrespective whether calculations based on facial (i.e. “true”) or mesial (i.e. typically radiographic) measurements.

## Materials and methods

An in vitro study design was chosen to establish definitive prospective crown-root ratios after simulated extrusion and crown lengthening procedures. Ethical approval was obtained from the local ethics committee at the Charité—Universitätsmedizin Berlin, Germany, at the Department of Prosthodontics, Geriatric Dentistry and Craniomandibular Disorders (approval number: EA1/034/06). Informed consent was obtained from all subjects and/or their legal guardian(s). This research was conducted considering the CRIS Guidelines (Checklist for Reporting *In-Vitro* Studies). Post-hoc power-analysis was performed with a free-to use software to calculate statistical power (G* Power 3.1.9.7; Heinrich-Heine-Universität Düsseldorf) with a sample size of *n* = 120, *α* = 0.05 resulting in a power of 0.99 (99%).

**Figure 2:**
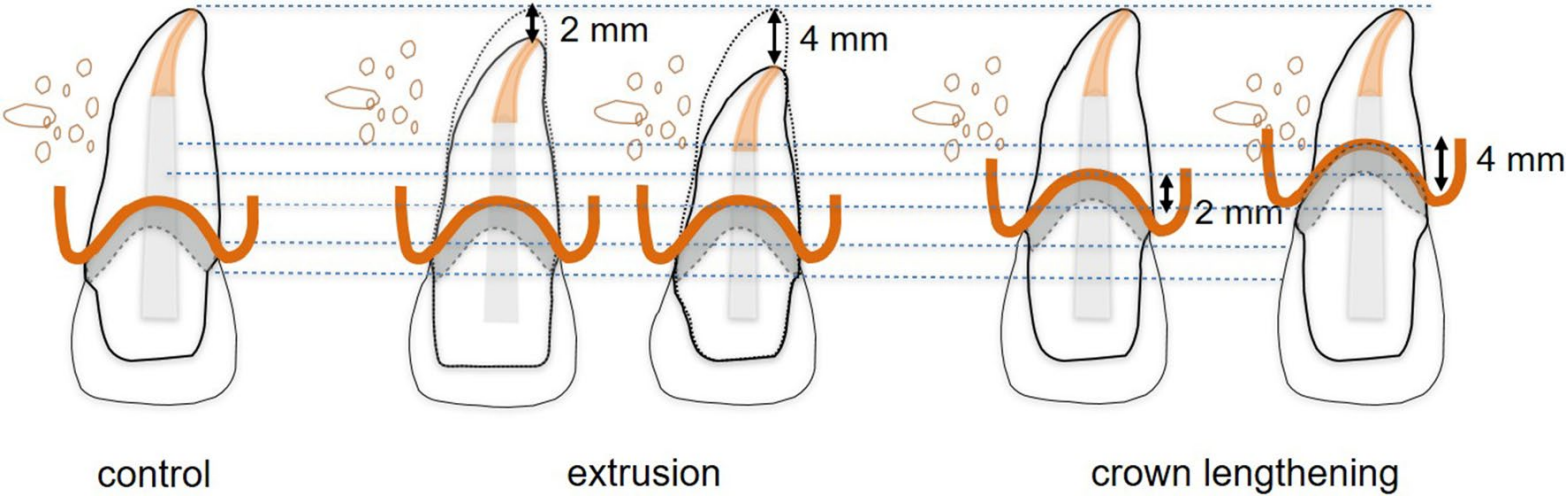
Overview of preprosthetic treatment measures: extrusion of 2 mm (E-2 mm) and 4 mm (E-4 mm) versus crown lengthening of 2 mm (CL-2 mm) and 4 mm (CL-4 mm).

128 human maxillary central extracted incisors were examined from a tooth reservoir at the Department of Prosthodontics, Geriatric Dentistry and Craniomandibular Disorders of the Charité —Universitätsmedizin Berlin, Germany. Maxillary central incisors with sound tooth structures were included. Teeth with root resorptions, restorations, carious lesions, erosive, and wedge-shaped defects were excluded. Figure 1 defines examined variables. Measurements were performed for 128 teeth and calculated for five experimental treatment groups (Fig. 2): C (control), E-2 mm (extrusion of 2 mm), E-4 mm (extrusion of 4 mm), CL-2 mm (crown lengthening of 2 mm), and CL-4 mm (crown lengthening of 4 mm). Tooth lengths (TL) in [mm] were recorded with the aid of a caliper gauge (HSL 246-15; Hammacher, Solingen, Germany) with a measurement error of 0.01 mm from the incisal edge to the apex of the tooth. The most apical point on the facial (f) aspect of the CEJ and the most coronal point of the mesioproximal CEJ served as reference points for measurement. Root and crown lengths (RL/CL) were measured from the mesioproximal and facial CEJ, respectively. Normal distribution was assessed based on RL-measurements. Assuming a biological width of 2 mm effective crown and root lengths (eCL/eRL) were calculated based on following formulas: eCL = CL + 2 mm and eRL = CL-2 mm. Thereafter, anatomic crown-root ratios (CRR) and effective crown-root ratios (eCRR) were calculated for all specimens. Mean values and standard deviations of measurements are displayed in Table 1. Descriptive statistics were performed with the aid of a statistical software (IBM SPSS Statistics 25; IBM, Armonk NY, USA). T-tests were performed for statistical comparison between groups. The significance level was set at *p* < 0.05.

**Table 1:** Tooth measurements and calculated crown-root ratios for extrusion and crown lengthening procedures.

	Control	E-2 mm	E-4 mm	CL-2 mm	CL-4 mm
TL [mm], mean ± SD	22.8 ± 1.6	20.8 ± 1.6	18.8 ± 1.6	22.8 ± 1.6	22.8 ± 1.6
CL-m [mm], mean ± SD	6.7 ± 1.1	6.7 ± 1.1	6.7 ± 1.1	8.7 ± 1.1	10.7 ± 1.0
CL-f [mm], mean ± SD	9.5 ± 1.0	9.5 ± 1.0	9.5 ± 1.0	11.5 ± 1.0	13.5 ± 1.0
RL-m [mm], mean ± SD	16.1 ± 1.4	14.1 ± 1.4	12.1 ± 1.4	14.1 ± 1.4	12.1 ± 1.4
RL-f [mm], mean ± SD	13.3 ± 1.3	11.3 ± 1.3	9.3 ± 1.3	11.3 ± 1.3	9.3 ± 1.3
CRR-m, mean ± SD	0.4 ± 0.1^a,e^	0.5 ± 0.1^a,f^	0.6 ± 0.1^a,g^	0.6 ± 0.1^a,h^	0.9 ± 1.7^a,i^
CRR-f, mean ± SD	0.7 ± 0.1^b,e^	0.9 ± 0.1^b,f^	1.0 ± 0.2^b,g^	1.0 ± 0.2^b,h^	1.5 ± 0.2^b,i^
eCL-m [mm], mean ± SD	8.7 ± 1.1	8.7 ± 1.1	8.7 ± 1.1	10.7 ± 1.1	12.7 ± 1.1
eCL-f [mm], mean ± SD	11.5 ± 1.0	11.5 ± 1.0	11.5 ± 1.0	13.5 ± 1.0	15.5 ± 1.0
eRL-m [mm], mean ± SD	14.1 ± 1.4	12.1 ± 1.4	10.1 ± 1.4	12.1 ± 1.4	10.1 ± 1.4
eRL-f [mm], mean ± SD	11.3 ± 1.3	9.3 ± 1.3	7.3 ± 1.3	9.3 ± 1.3	7.3 ± 1.3
eCRR-m, mean ± SD	0.6 ± 0.1^c,e^	0.7 ± 0.2^c,f^	0.9 ± 0.2^c,g^	0.9 ± 0.2^c,h^	1.3 ± 0.3^c,i^
eCRR-f, mean ± SD	1.0 ± 0.8^d,e^	1.3 ± 0.2^d,f^	1.6 ± 0.3^d,g^	1.5 ± 0.2^d,h^	2.2 ± 0.4^d,i^

*SD* standard deviation, *E* extrusion, *CL* crown lengthening, *TL* tooth length, *CL* crown length, *RL* root length, *CRR* crown-root ratio, *e* effective, *m* mesial (measurement from the mesial CEJ), *f* facial (measurement from facial CEJ).

Identical letters indicate significant differences at *p* < .05: first letter shows group differences within a row, second letter within a column.

## Results

**Figure 3:**
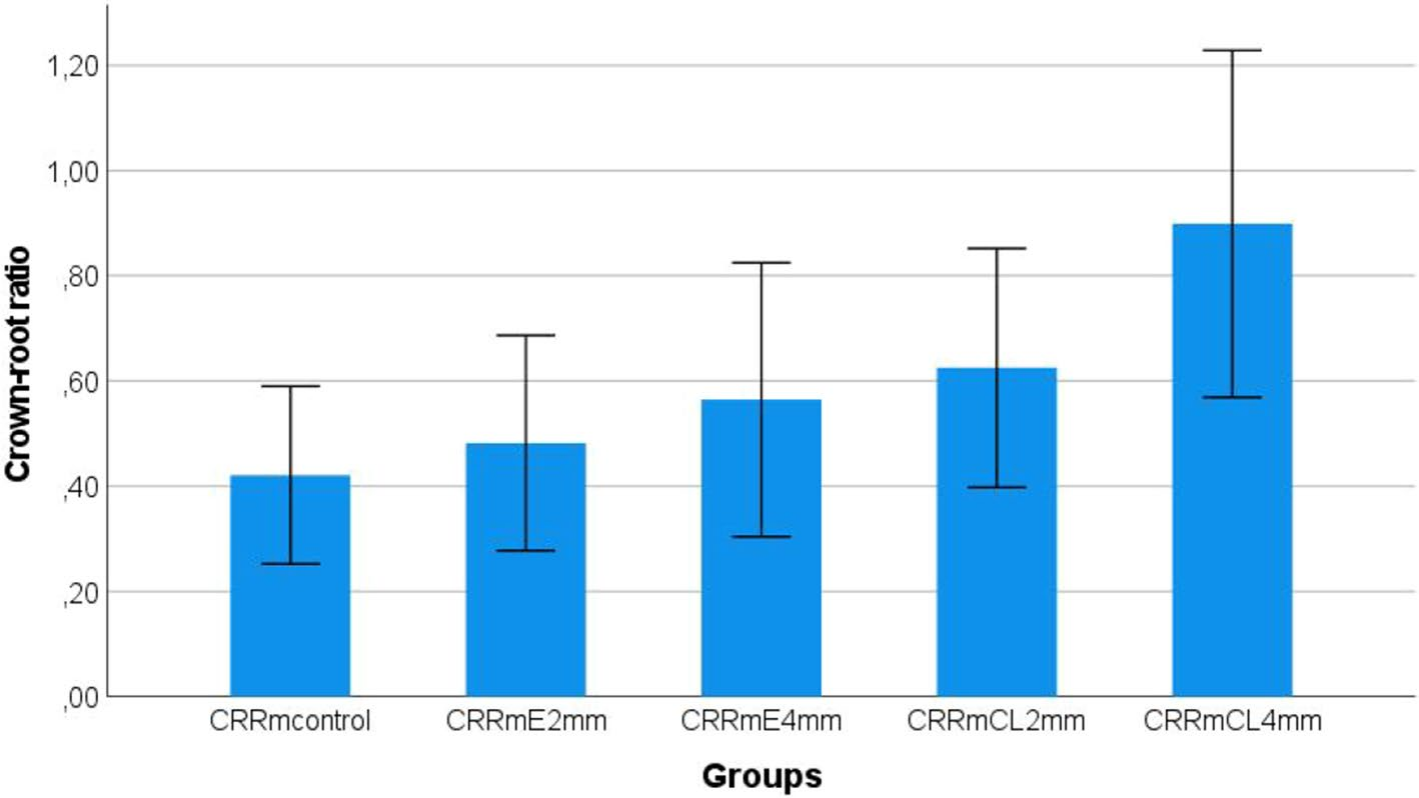
Anatomic crown-root ratios for experimental treatment groups with mesioproximal cemento-enamel junction as reference. CRR = crown-root ratio, m = mesial, E = extrusion, CL = crown lengthening.

**Figure 4:**
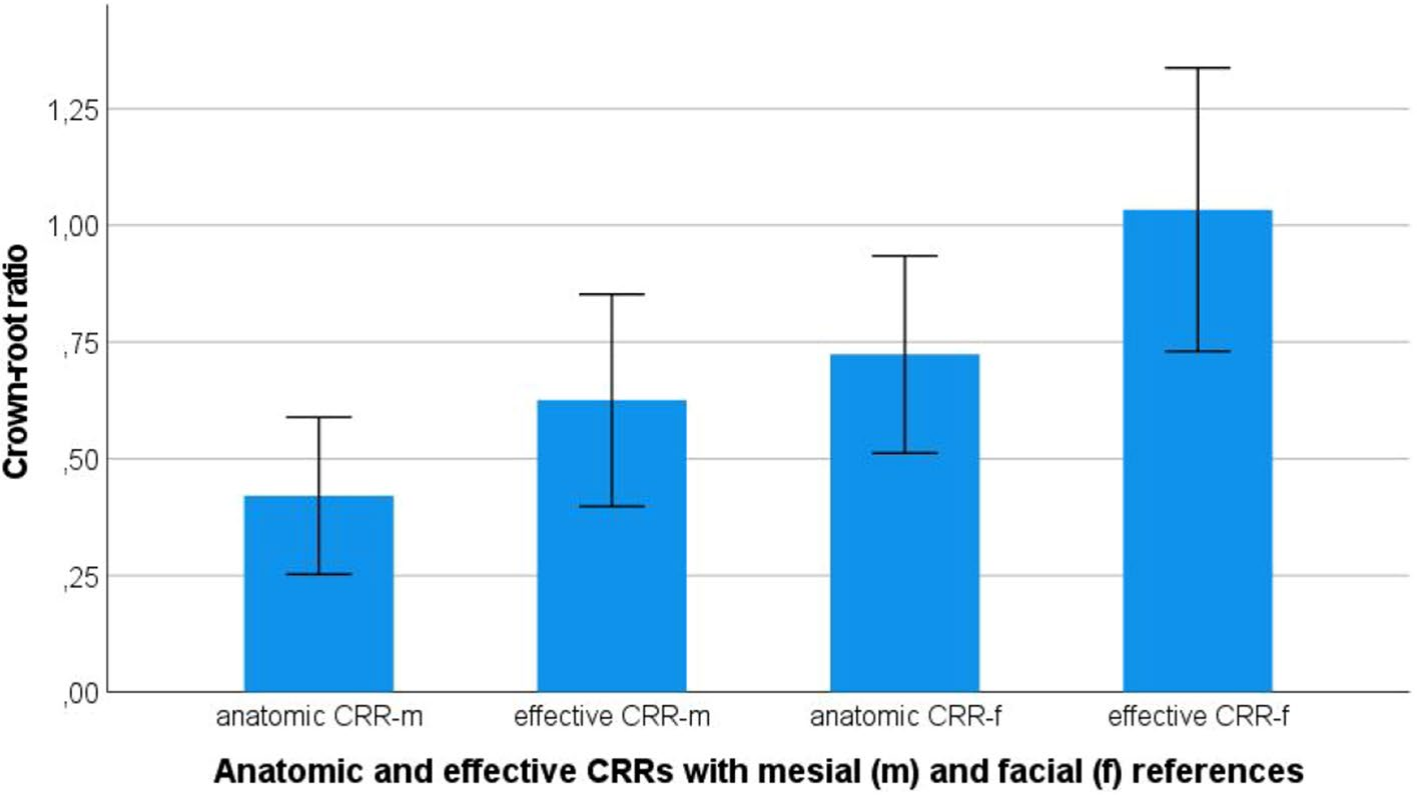
Differing crown-root ratios (CRRs) in dependance of measurement method for control group.

Root lengths (RL) of 128 teeth were measured. Eight teeth were excluded from further analyses due to their extreme short and long root lengths, respectively. Table 1 summarizes tooth length (TL), crown length (CL) from facial (f), and mesioproximal aspect (m), root length (RL), effective measurements (e), and resulting crown-root ratios (CRR). For maxillary central incisors, the following mean [± SD] anatomic crown-root ratios (CRR) and effective crown-root ratios (eCRR) were recorded: CRR-m = 0.4 [± 0.1], CRR-f = 0.7 [± 0.1], eCRR-m = 0.6 [± 0.1] and eCRR-f = 1.0 [± 0.8]. Comparing measurements for extrusion of 2 mm with crown lengthening of 2 mm all crown-root ratios are lower for extrusion (*p* < 0.001). For extrusion of 4 mm (E-4 mm) and for crown lengthening of 4 mm (CL-4 mm) all crown-root ratios are lower for extrusion with *p* < 0.001 (Fig. 3). Values for CRRs were significantly different between all theoretical treatment groups (*p* ≤ 0.001). Thereby, effective crown-root ratios were higher than anatomic crown-root ratios. CRR referring to mesial CEJ was lower than CRR referring to facial CEJ (Fig. 4).

## Discussion

This is the first study that provides “real-life” and exact crown-root ratios after measurement of 120 extracted teeth and compares extrusion with crown lengthening procedures for the assumed clinical case of a missing clinical crown of a maxillary central incisor, i.e. the tooth crown is missing at a gingival level. It was shown, that mesial measurement-based calculations of crown-root ratios typically based on radiographic images should be interpreted with caution as they underestimate (appearing as more favourable) the effective crown-root ratio. Prospective crown-root ratios are lower, i.e. more favourable for extrusion than for crown lengthening procedures. Therefore, both null hypotheses were rejected. The mean effective crown-root ratio measured at the mesial CEJ for maxillary central incisors without preprosthetic treatment measures was 0.6 and the mean effective crown-root ratio measured at the facial CEJ was 1.0, respectively. The eCRR for 2 mm of extrusion was 22% lower compared to the crown lengthening group of 2 mm. For 4 mm of extrusion eCRR was even 30% lower than in the crown lengthening group. Hence, with an inevitable lengthening of the clinical crown due to crown lengthening to enable a reconstruction of the damaged tooth, the crown length is increased while the effective root length, i.e. the part of the root supported by alveolar bone, is decreased. Thus, two parameters have a potential negative impact on the crown-root ratio. In contrast, extrusion changes only one parameter. The effective crown length remains constant, while effective root length decreases. Therefore, in regard to the resulting prospective crown-root ratio extrusion may be favoured over crown lengthening^14,15^.

The study investigated maxillary central incisors as this region was described as “high risk” area for mechanical failure^28^ and is functionally and aesthetically of utmost interest. In the anterior region non-axial shear forces occur, whereas in the posterior region axial, compressive forces are more likely. Therefore, anterior restorations are much more susceptible to technical complications.

However, it is one of the limitations of this in vitro study that solely maxillary central incisors were investigated. Crown-root ratios of premolars and molars as well as maxillary and mandibular incisors may be different. Moreover, based on the present results the impact of CRR on long-term clinical success of restorations still remains uncertain, since additional factors play a role. Further, surgical crown lengthening procedures may be contraindicated in the anterior region for aesthetic reasons as they are accompanied by a lengthening of the clinical crown, and an osseous reduction of the alveolar bone is disadvantageous in the context of a possible prospective implant placement. In contrast, for orthodontic extrusion procedures a tendency for marginal bone gain has been observed in a clinical pilot-study investigation^29^.

Tooth, crown, and root lengths in this study are in line with published data^30^. They can therefore serve as a reliable basis for calculation of prospective crown-root ratios. Data show that crown-root ratios are highly dependent on measurement methods. The study presented four different measurements with different reference points for the crown-root ratio: crown-root ratios ranging from 0.4 to 2.2, respectively. They are higher if measured from facial in comparison to mesioproximal CEJ in all groups, which would be typically calculated based on X-rays. Effective crown-root ratios exceed a value of 1 in all experimental groups, if measured facially. Data show that crown-root ratios measured at the mesial aspect account to nearly 60% of the crown-root ratios measured at the facial aspect. This finding leads to a miscalculation of the “true” crown-root ratio as the lever arm is much higher than assumed prior to restorative treatment decisions. However, the anatomic “true” crown-root ratio measured from facial aspect may not be determined clinically nor radiographically. Ultimately, the only clinical possible measurement method for determination of CRR is the e-CRR-m as it may be determined radiographically at the proximal marginal bone level^31^. Therefore, measurements of crown-root ratios based on radiographic images should be interpreted with caution as they underestimate the anatomic crown to root relation.

Scientific evidence on the impact of specific crown-root ratios on biomechanical behaviour is rather low: An in vitro study has demonstrated a reduction of static load failure for decoronated mandibular second premolars after crown lengthening procedures and in presence of a ferrule^20^. Results are in accordance with another in vitro study showing that extrusion method reduced the crown-root ratio fewer and increased the root-fracture resistance more than crown-lengthening method^26,27^. However, studies are missing to establish distinctive proportions leaving the general practitioner with vague terms as “unfavourable”, “favourable”, “poor” and “good”. Moreover, clinical studies on the biomechanical impact of altering crown-root ratios are scarce, especially in regard to teeth that underwent extrusion. A review has attributed to a lack of clinical data on the impact of crown-root ratio on long-term prognosis in general^23^. One of the few longitudinal clinical studies investigated 236 clasp-retained removable partial dentures and found a significant risk for abutment failure with a crown-root ratio exceeding 1.0^24^. Other authors also found a positive correlation between the crown-root ratio and long-term prognosis for patients with periodontitis under maintenance for 5 years^32^. A clinical study evaluated the impact of the crown-root ratio on the 10-years survival rate of endodontically treated teeth after surgical crown lengthening and concluded that an “inadequate” crown-root ratio exceeding 1.0 has a negative impact on long-term survival^25^. For orthodontic extrusion long-term clinical data are scarce and mostly limited to case reports and case series^13,16,17^. Studies, both in vitro and in vivo have to evaluate the biomechanical impact of altering crown-root ratios on both treatment concepts in future.

## Conclusions

While for extrusion only root length is reduced (crown length unaltered), for crown lengthening root length is reduced as much as crown length is enlarged. Hence, prospective crown-root ratios are lower, i.e. more favourable for extrusion than for crown-lengthening procedures. Mesial measurement-based calculations of CRR based on radiographic images should be interpreted with caution as they underestimate (appearing as more favourable) the effective CRR.

## Data Availability

The data presented in this article are available from the corresponding author upon reasonable request.
